# The Preparation and Filling of Roots at One Sitting

**Published:** 1890-11

**Authors:** E. L. Clifford

**Affiliations:** Chicago.


ARTICLE III.

### THE PREPARATION AND FILLING OF ROOTS AT ONE SITTING.

BY. E. L. CLIFFORD, D. D. S., CHICAGO.

An article in "Items of Interest" under the caption, "I do not indorse immediate root filling," reads as follows: "We can never know that incipient abscess is not present, and, if it is, trouble will always follow such an operation. It is to be deplored that this practice is advocated to such an extent in our periodical literature, on account of the danger of leading young men astray. We should be more conservative in our practice. A root may be filled immediately if we have destroyed and removed the pulp ourselves, but, even in those cases, I would prefer to leave it a day or two, to destroy any living tissue that might remain in the canal. For this purpose I know of nothing better than carbolic acid .95 per cent.

The reason given for the position taken in the above paragraph, and the only reason, is, "We never know that incipient abscess is not present." Well, suppose it is present? What is an "*incipient*" abscess? In medical lexicology I have hunted in vain for an interpretation. The term incipient, I know, is used with freedom by medical writers, and will therefore exonerate the authority quoted from any intention to launch a new word upon our already crowded sea; but as the term has not been thought of sufficient importance to find a place in our special lexicons, I take it for granted that it is used simply in its original and literal meaning. Therefore the term incipient abscess must mean an abscess just beginning, for the word is defined, "beginning, commencing, starting." Now, what has pathology taught us is the beginning of all abscesses? Is it not inflammation? And must not inflammation be preceded by, first irritation, then contraction and dilatation of the vascular surroundings, followed by hyperæmia and more or less complete blood stasis, to be in turn followed by the various steps consequent upon any inflammation; and let us not forget, at this point, that inflammation does not *necessarily* lead downward through the grade of retrograde metamorphosis to finally reach suppuration, atrophy, gangrene, or death. But remember that life is tenacious; that physiological tendencies, with equal fortune, will invariably overcome and overbalance pathological drift, the inclination of all tissues to return to the normal health status being so great. No. "Inflammation ceases to advance just as soon as the irritation is removed, and the blood, circulating through the vessels, restores their walls to a healthy state. When this happens, *recovery* at once begins. The injury to the vessel-walls being slight, and the exudation trifling, the vessels soon begin to perform their normal functions, and what is effused undergoes reabsorption by the lymphatics, or by the blood vessels themselves."

Now, then, this fact established, should the presence of an incipient abscess debar us from finishing our operation, *if* it has been possible to thoroughly clean and sterilize every portion of the pulp canal and dentinal tubuli, and can successfully reach with our filling material the apical foramen. I think not. The position I take in regard to devitalized teeth is, that there is but *one* object to be accomplished in their preservation; that is to *clean* the roots; and this I believe can, in a majority of cases, be done (I will not say as well), at one sitting than twenty. In using the word clean, I, of course, use it in its most superlative sense, no half way, or "that is well enough," should ever satisfy the honest operator.

If, then, our sole object is to clean the root, it signifies that the root contains something that is unclean and impure. The question then arises, if this unclean and impure matter is contained in the root in what portion of the root does it find a habitat and a lodgment? Is it in the pulp canal, and the pulp canal *only?* If so, it would be an easy matter to purify and clean all roots presenting, at one sitting. But are we not stubbornly met with the fact that the boundary line of this unclean substance, and its effects, reaches farther than the above suggestion, and therefore that our remedies must penetrate farther to be effectual.

Another question would here naturally arise: Can we follow it, and if so, how? Another digression forces itself upon me at this point, but only for a moment, as I will accept it without comment; that is, to accept the germ theory as an etiological factor in the pathological condition to be overcome. I take it for granted that micro-organisms are accepted as prime movers in all pyogenetic affections, at least until some other theory more plausible is advanced; and from this bulwark will fire the random shots destined for the field of enquiry upon the ground of progressive surgery. Fermentation and putrefaction having advanced sufficiently, the habitat or lodgment of the micro-organisms has, of course, become too small; their products have generated and produced sufficient gaseous substance to more than fill the cavity of the pulp chamber and canal; consequently a hunt for other quarters is their ultimatum. The nearest and most direct outlet we believe they will take and our minds are at once directed to the dentinal tubuli and apical foramen. The tubuli, having been occupied by animal matter, must certainly come in for their share of fermentation and putrefaction and their results, and must so harbor nests of micro-organisms that might revolt at any moment, declare an insurrection, and proceed to wage a merciless war upon the surrounding tissues. For this reason, it will readily be seen that it is not enough to clean and sterilize the root canal proper, but these tubuli must also be emptied, cleaned, dried, sterilized and filled.

As to how this shall be done: My first thought in the procedure of such an operation is, *thorough dryness and complete* protection from any future inundation of saliva, water, or other septic fluid. To gain this, the rubber dam is brought into play, safely adjusted, and the cavity of decay and pulp chamber relieved of its detritus. Having removed all possible with spoon excavators, and dried as much as possible with bits of amadou, I then saturate the chamber with a *non*-escharotic disinfectant, waiting a few moments, again dry the cavity, and proceed to remove any particles of putrescent pulp within the canal. Should pus already have formed, I use alternately injections of peroxide of hydrogen and an ethereal solution of iodoform, believing that, with these agents, I simplify and render easy what would otherwise be tedious and difficult. I do not know that I have seen a reason given for this alternating of $H_2O_2$ with iodoform and ether, but, believing that to be scientific, we should have an end to accomplish in each step taken to re-establish a physiological function, or to eradicate a pathological condition I will try to give you my reasons for each step taken.

First, then, we court perfect dryness. Why? Because moisture, warmth and microbes are the three essentials to septic fermentation. Absence of any *one* of these is sufficient to prevent decomposition.

Second, protection from saliva. Why? First, because it is a liquid, and its presence would thwart our efforts at dryness; next, our scientific researches have shown us that saliva contains and propagates, not only all classes of bacteria now known to us, but most of the fungi as well. Not only benign micro-organisms find a flourishing element in saliva, but most of the pathogenetic species also. In fact, nearly fifty separate and distinct foreign and malignant substances have been extracted from the human saliva, ranging from a simple mucous corpuscle to pus corpuscles and all the malign bacilli known to exist within the animal economy.

Third. Why disinfect the cavity of decay? That you may not push into your pulp chamber, and from thence into and through your root canals, the septic matter you know to exist in that habitat; also on the principle of

"an ounce of prevention being worth a pound of cure."

So far we have had a comparatively easy task. We have had only that portion of our root to deal with that we could see or feel. If, however, putrescence has taken place, disintegration has, of course, been established, and it will be impossible to remove the pulp entire, and the process of removing it mechanically is too long and tedious, (especially if the case in hand has more than one root.) What, then, shall be done? It is my practice, after removing all possible in the manner described above, to saturate the chamber and canal with the volatile extract of eucalyptus (made by Sander & Sons, of Australia; I never use any other), and then drying with amadou, to apply chloroform to saturation. My object in doing this is two fold: First, for its analgesic effect; but mostly as a solvent for the oils and fats left adherent to the walls of the canal, and occupying the spaces of the tubuli.

(Although not pertinent to the subject in hand, I will state that if I were not going to fill at one sitting, and wished to accomplish the same end by prolonged treatment to two or more sittings, I should fill the pulp chamber and canals with Squibbs' carbonate of soda, and leave it for three days. I should thereby saponify these oils, fats and extraneous matter with this alkali, and, at the end of the time named, a little warm water would be all required to wash out and clean the root.)

The fatty substance being disposed of, in what condition do we find our organ? Tolerably clean, but we have been compelled to use liquids; therefore we still possess one of the main elements necessary to the growth and propagation of pathogenetic material; *i. e.*, moisture. We insert our cotton, bibulous paper, amadou, and all the other absorbents at our command, but still our work is defective; it is not complete; we have not been thorough, and there is only one element in nature known to me that can be called to our assistance. That element is heat; and although the statement has been made that heat as an antiseptic was useless to the dentist, from the fact that some scientist had discovered that 212° F., and that maintained for 1½ hours, was necessary to destroy microbes without spores, and that the heat must be increased to 284° F., and maintained from 1½ to 3 hours to destroy the spores. I am satisfied in my own mind, and will endeavor to show, that we do not use heat for its direct antiseptic effect; but it is one of our best assistants towards that end. The statement has also been made, that the perfect drying of a root by heat (such, of course, as can be used in the mouth) will not destroy germs or spores. To all this I bow in humble acquiescence. But, acknowledging the fact, is there no other assistance that heat can render us in accomplishing this end? After drying as well as possible with our absorbents, if we can contrive some method or appliance by which we can carry heat much greater than the normal surroundings of the root into the canal, we certainly can extract from its hiding place all the moisture possible. We certainly know it is a law of physics that no two substances can occupy the same space at the same time. We also know that a superheated instrument, coming in contact with water, will convert it into steam, which is gaseous, and cannot be confined, but must escape; vacuum must be the result. We also know that nature abhors a vacuum, and that these osseous structures, having been deprived of their natural quota of water will seek for and find, if possible, its requisite. Taking advantage of this physical law, we supply the new moisture, first charging that moisture with some element known to be antagonistic to growth and development of pathogenetic elements. I select the ethereal solution of iodoform. The liquid penetrates into all the anfractuosities and diverticula of the bone or abscess, the ether becomes absorbed or evaporated, and the agent is deposited uniformly on the pyogenic membrane, the action of which it modifies, dissolves any oils or fats which may have been left in the tubuli, and leaves the spaces filled with the medicament used. Now, having the root thoroughly saturated with iodine, I promote further evaporation by warmed air, and take a further advantage of our physical law of suction, and compel the dentine to attract chlora-percha solution or oxy-chloride of zinc, whichever material I am using. Of course the less dentine we have in the roots the less disinfecting we are called upon to accomplish; and, if the strength of the organ will permit it, and your sense of touch is sufficiently skilled, I would admit the theory of Dr. Allport, and advise the removal of as much dentine as possible. I should advance, however, in the practice of this theory with caution.

And now to review—are there any impossibilities stated in the foregoing, are there any illogical deductions drawn? You will all recognize the fact that these several steps can be followed in less time, or as much at any rate, as the detailing of them will require, and why shall a patient be required to spend from three days to three weeks in running to our offices when the end can be accomplished in one hour of continuous work. A case in practice comes to mind—a *confrere* resident in one of our suburbs had for a patient a lady about thirty years of age who lived on the west side and consequently a great distance from his office. One of the lower bicuspids had abscessed and the patient had spent her time and money for several months in semi-weekly visits to her dentist with the result that each time the tooth was tightly sealed the inflammation would reappear. The dentist told her that if she had any trouble after his last treatment to call on me. It was only a few days when the patient applied to me with a terrible sore tooth and considerable pain. After getting a full history of the case I removed the dressing in the root, found considerable suppuration, and after washing out as much as possible I filled the canal with carbonate of soda, and dismissed the patient for three days. (You will recognize that I was dealing with another man's patient and felt that I would take no risks so I gave two treatments). At the appointed time she returned, when the saponified residue was washed out and a line of treatment similar to what I have detailed above was pursued. The root was filled with oxychloride of zinc and hydronapthol (at that time I had not used chlora-percha) and the crown filled with oxy-phosphate, with instructions to return to her dentist at the end of two weeks and have a gold filling inserted.

Another case—A lad about 18 years presented r. l. bicuspid terribly decayed, jaw considerably swollen, but as he lived within a few doors of my office where I could watch him I wanted him for an experimental case. He wanted the tooth extracted. It was in the forenoon and I had a patient in the chair, so I opened into the pulp chamber when his mouth filled with pus and he was to some extent relieved; I told him to call again at 2 o'clock, which he did, when I adjusted the rubber dam, cleaned and filled the roots and crown. It is now about five months and I see the patient often and he is delighted that he did not have the tooth extracted. Now the question may very properly be asked, from what I have stated, if I would advise the filling of *all* roots at one sitting. Judgment must of course be used in any and all operations of surgery, no matter how trivial they may appear. But I am willing to be placed upon record with the statement that the conditions forbidding the procedure are the exception by far and not the rule. I believe the fact is established, and one upon which there is no difference of opinion at this time, that all aseptic canals are ready to be filled. I, also, believe the fact established that all canals possessing an exit through a fistulous opening in the gum may as well be at once cleaned and filled. Thus you will see these two facts would probably embrace three-fourths of all the canals we are called upon to fill, leaving a small minority about which there would be question. In reflecting upon this minority the principal reason, usually given, that would negative our procedure would be the fact of no existing fistula, or what is generally termed a blind abscess. This fact *alone* I cannot accept as reason sufficient for protracted treatment, as experience has shown that (though possibly provoking an acute pericementitis for twelve hours or more) in a vast majority of cases the great tendency of nature to reassert herself and the physiological proclivities being so much greater and overpowering the pathological in her effort to return to health, that victory will at last be proclaimed upon the right side, and a complete subsidence of all outward manifestations will be the result. Should I fear however that the vital energies of my patient may be taxed too greatly, I always anticipate and relieve the immediate pressure by establishing what nature has thus far failed to make, an artificial fistula, and prescribing a few doses of some of our newer anti-neuralgics and analgesics.

From the above, what would we conclude to be the essentials to success in root filling? First, perfect dryness; second, no vacuum; and third, as precautionary and preventive, drainage, as in all surgical cases in other portions of the organism. I can say, with the editor of the *Western Dental Journal*, that immediate root filling should not be practiced unless dryness, which is the first step toward an aseptic condition, can be secured; and this condition I believe can as well be secured in blind abscess as in those with a fistulous opening (hæmorrhage excepted). So much so am I convinced of this fact, that I have reduced the conditions to two that would prevent and forbid an attempt to immediately fill a root presenting for treatment. One of those conditions, and the main one, is the impossibility to dry the whole of the dentine. This will be the case at times in persons of a hæmorrhagic diathesis. The other where the tissues are so swollen and painful as to render it cruel to operate at that time. The treatment I can but regard as philosophical and logical. It is but a sequence of a proved knowledge so beautifully illustrated to our society by Dr. Andrews, of Cambridge, at our recent clinic, that the disease is the result of sepsis, and when the septic condition has been removed, and its place taken by an impermeable and indestructible filling, a certain cure must result, just as certainly as if the canal had been obliterated by the extraction of the tooth. Any remaining products of the once-existing irritant will speedily be absorbed and changed into healthy cicatricial tissue. One condition may exist that would retard and probably prevent a subsidence of all irritation, and that would be the presence of necrotic tissues either in the root or maxilla. In the successful treatment of this condition, however, the passage through the root is not essential, and would not debar me from filling the root, as I would continue the necessary treatment through the gum, and expect, in time, a recovery.

The question also arises, if it is deemed hazardous to immediately fill these canals, why is it? What would result? What could result? Well, although the past life of this subject is short, there is hardly any pathological condition that has followed an operation that has not been attributed (not to the filling of the root, not to the existence of an almost foreign substance embedded in the tissues, not simply to dental irritation, but) to *immediate* root filling. A case is reported in the February number of the *Archives*, in which considerable mischief is said to have resulted from *immediate* root filling. The reporter of that article starts out by saying that the patient was served by a *fine* operator, and winds up the facts of his statement with the remark that "the other tooth was *reamed out* and filled." Well, if this reporter has told the whole truth, and nothing but the truth, about the operation, I do not see how any but unfavorable results could be expected. *Reaming* out and *digging* out are certainly not very euphonious terms to the professional ear, and certainly cannot be said to constitute the necessary steps to any surgical precedure; so that, if the statement can be borne out by the facts, the results were certainly not from *immediate* but from *imperfect* root filling, which may have occurred in any case treated for months.

As stated before, almost all varieties of reflex and disseminate neuroses have been attributed to this operation.

You all remember a case presented to this society only a few meetings ago, and reported as a result of immediate root filling—a case of alopecia areata. Now, although in the highest degree probable that alterations, and particularly shedding of the hair, may at times be dependent upon nervous influence, yet exact proof of this fact is still wanting. The affection, according to late investigators, is considered as a tropho-neurosis. Experiments have been made, and clinical observations recorded with reference to the influence of disturbances of nutrition, and also of psychoses, upon the condition of the hair; but the data so far obtained is extremely deficient. Joseph has asserted that such a thing as a distinct bundle of trophic nerves does exist, and also that he can produce a circumscribed alopecia in cats by the extirpation of the spinal ganglia of the second pair of nerves of the neck, together with portions of the neighbouring posterior and anterior roots. While these observations thus far made, taken singly, would not be convincing, yet, viewed collectively and in connection with the physiological experiments, we seem to have strong evidence of the tropho-neurotic nature of circumscribed alopecia. Hence it would appear that any irritation reflected to the trophic centres might result in this condition, especially so when we notice that headache is one of the commonest premonitions of alopecia. Cases have also been reported in which heredity was an etiological factor in alopecia areata; and when the neuropathic predisposition is present, very slight influences are enough to cause the appearance of some nervous disease. The most prominent causes, however, are the traumatic and the psychical, and it is not astonishing to find the disease following either of these causes, when we reflect that it is only the merging of a hereditary proclivity. The question of age has also entered into the inquiries upon this subject, and 12½ years was found to be the average at which the disease appeared; so it has been suggested that it is in some way connected with the second dentition and puberty; but the fact is not established, and at present we are only certain of one condition dependent upon the trophic nerves, and that is the suspension of their activity, giving rise to subsequent atrophy.

The foregoing may indeed seem a digression to you, but it has proved interesting to me to collect these facts, in order that we might more thoroughly understand the etiology of a pathological condition reported to us as a result of immediate root filling.

I am satisfied the reporter of the case did not intend to convey the impression even that the disturbance was caused by the abstract fact of *immediate* root filling, but that it was the effect of an ill-judged dental operation, resulting in a dental irritation, reflected through the sympathetic ganglia to the trophic centres, and arousing in the case presented, a predisposed hereditary proclivity. I have too exalted an opinion of the diagnostic powers of my friend, not to take this view, especially as I could report another case similar of a young girl who glories in the possession of two bald spots, about the size of a silver dollar, upon the right parietal region, which ceased to enlarge, and again began to be supplied with a new growth of hair after the extraction of an upper molar tooth (which had never been filled) and a few visits to the Sutherland sisters.—*Dental Review*.

